# Radiosurgery with photons or protons for benign and malignant tumours of the skull base: a review

**DOI:** 10.1186/1748-717X-7-210

**Published:** 2012-12-14

**Authors:** Maurizio Amichetti, Dante Amelio, Giuseppe Minniti

**Affiliations:** 1 ATreP, Provincial Agency for Proton Therapy, via Perini 181, Trento 38122, Italy; 2 Neurooncology Unit, IRCCS Neuromed Institute, VIA Atinense, Pozzilli, (Isernia) 86077, Italy; 3 Sant’ Andrea Hospital, University Sapienza, via di Grottarossa, 1035, Rome 00189, Italy

**Keywords:** Stereotactic radiosurgery, Protons, Skull base

## Abstract

Stereotactic radiosurgery (SRS) is an important treatment option for intracranial lesions. Many studies have shown the effectiveness of photon-SRS for the treatment of skull base (SB) tumours; however, limited data are available for proton-SRS.

Several photon-SRS techniques, including Gamma Knife, modified linear accelerators (Linac) and CyberKnife, have been developed and several studies have compared treatment plan characteristics between protons and photons.

The principles of classical radiobiology are similar for protons and photons even though they differ in terms of physical properties and interaction with matter resulting in different dose distributions.

Protons have special characteristics that allow normal tissues to be spared better than with the use of photons, although their potential clinical superiority remains to be demonstrated.

A critical analysis of the fundamental radiobiological principles, dosimetric characteristics, clinical results, and toxicity of proton- and photon-SRS for SB tumours is provided and discussed with an attempt of defining the advantages and limits of each radiosurgical technique.

## Introduction

The skull base (SB) forms the floor of the cranial cavity and separates the brain from other facial structures. It is a very complex anatomical region that includes portions of the anterior cranial fossa, clivus, petrous bone, middle cranial fossa, cavernous sinus and infratemporal fossa encompassing several critical neurovascular structures.

Many histologic tumour types arise in the SB. They are rare lesions representing a very heterogeneous group from benign to malignant tumours.

Skull base tumours are challenging lesions because of their anatomical location. Surgical intervention is often the first step in therapeutic management, allowing for pathologic analysis with complete or partial tumor removal. Due to the proximity of critical normal structures, surgical intervention can result in complications that severely affect vision, hearing, speech, swallowing, and could even be life-threatening.

New radiation therapy (RT) techniques allow targeting SB tumours when surgery is not feasible, macroscopic residual is left after surgical intervention or as an alternative, definitive treatment. Most patients with lesions of the SB have a benign tumour and are expected to live for an extended period. Due to the close proximity of multiple critical normal structures, highly conformal RT techniques are desired to achieve a steep dose fall-off at the edge of the target volume, decreasing dose to surrounding structures in order to reduce the potential morbidity of treatment.

Recent technological advances in photon-RT have allowed improved targeting accuracy. External beam radiation therapy (EBRT) using conventional fractionation delivers a total radiation dose in multiple daily sessions of 1.8 - 2 Gy, five times a week to a histology-dependent total dose. Contemporary EBRT for SB tumors typically involves both image-guided intensity modulated radiation therapy (IMRT) and conventional fractionation stereotactic radiotherapy (FSRT) to maximize target conformality and reduce dose to adjacent normal structures. Conventional fractionation is usually reserved for lesions larger than the ones usually treated with single fraction radiosurgery, or for tumors abutting or compressing critical normal structures where there is no adequate separation for single fraction treatment. Radiosurgery options include:

1. Stereotactic radiosurgery in a single fraction (SRS): irradiation is delivered by a single large dose with very steep fall-off in dose outside the lesion to small volumes in order to be tumouricidal or ablative [1],

2. Hypofractionated stereotactic radiation therapy (HSRT): multi-session radiosurgery (two to five sessions) delivering larger doses per fraction than EBRT but not as large as with SRS.

Different techniques are used to deliver stereotactic treatments, utilizing precise patient immobilization, set-up uncertainty reduction, targeting accuracy, delivery of high doses, and heterogeneous dose distribution with a steep dose gradient. The tumour volume to be treated is crucial, and some have advocated HSRT for large-volume tumours. In these cases, target conformality and selectivity are reduced in comparison to SRS. At present, there are no long-term data to substantiate the role of HSRT over SRS for appropriately-selected patients. Preliminary data [2] suggest that HSRT may represent an effective treatment associated with lower risk of radiation-related adverse effects in patients with perioptic or large benign tumours as compared to SRS, although potential benefits remain to be demonstrated. For the purpose of this review, from now on our discussion will focus only on SRS showing technical characteristics and clinical results of its application.

**Figure 1:**
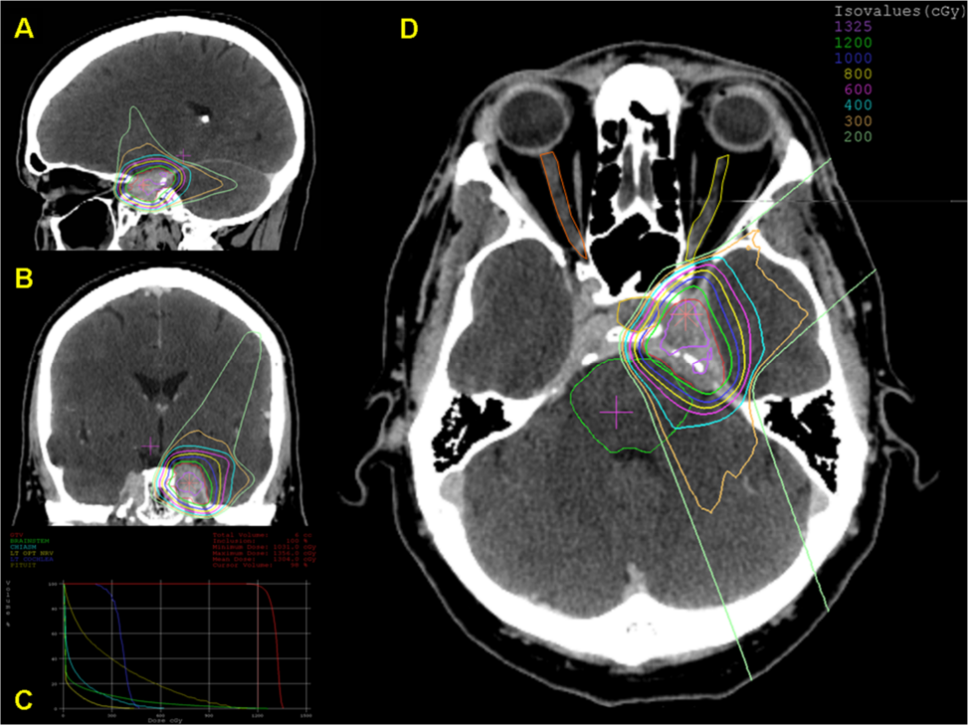
**Dose distribution in sagittal (A), coronal (B) and axial (D) views of a left cavernous sinus benign meningioma treated by proton radiosurgery**. Tumour volume was 5.7 cubic centimetres (in red). The treatment was delivered with a dedicated radiosurgical device (STAR). A dose of 12 cobalt gray equivalent (CGyE) was prescribed to 90 per cent isodose. Three equally weighted passive scattering beams were employed. The dose-volume histogram graph (**C**) shows the doses to organs at risk (optic chiasm in sky-blue, left optic nerve in bright yellow, brainstem in green, left cochlea in blue, pituitary gland in dark yellow) and tumour volume (in red). Courtesy of Francis H. Burr Proton Therapy Center – Massachusetts General Hospital, Boston (USA).

Despite the enhancements in delivery with better conformality indices, photons still have a relatively high exit dose (beyond the tumour target), which can produce significant normal-tissue exposure. Protons have similar biological effectiveness to conventional photon radiation but the defined range exhibited by the Bragg peak results in an energy deposition with no exit dose beyond the target volume. These fundamental physical properties enable proton RT to offer superior dose distribution and reduced low-dose integral irradiated volume [3], allowing more radiation dose to be delivered to the tumour while significantly lowering the dose to the surrounding normal tissues (Figure 1).

Protons appeared very attractive as a stereotactic radiation tool mainly in their developmental phase when many of the comparisons for the advantageous dose distributions were made against simple photon field arrangements, which are now obsolete.

Protons have a dosimetric advantage over photons, particularly in the case of larger intracranial lesions. However, as the smaller lesions are concerned, alternative techniques developed in the meantime, such as Gamma Knife (GK) and linear accelerators (Linac), appeared to be equally effective and less costly.

Considering the continuous advancement of technology in delivering SRS with photons and the increased use of protons, we have deemed it useful to review this issue by evaluating differences with photons and possible advantages of the use of protons as a radiosurgical technique.

### SRS delivery systems

Photon stereotactic irradiation can be delivered with different machines and techniques: Cobalt-60 gamma radiation-emitting sources introduced in clinical practice at the end of the 60’s or Linac adapted for radiosurgical works available from 1980.

#### Gamma knife

In its mostly used version, GK contains 201 small Cobalt-60 sources of gamma rays arrayed in a hemisphere within a thickly-shielded structure. A primary collimator aims the radiation emitted by these sources to a common focal point. A second external collimator helmet has an array of removable tungsten circular collimators of different sizes (one per source) that are used to create different diameter fields at the focus point. Patients are typically immobilized in a fixed frame with a positioning accuracy <0.5 mm. The dose is typically prescribed at 50 per cent to obtain the maximum dose at the centre of each pinpointed target and minimal dose at target edge. For large, non-spherical targets, like the majority of SB tumours, a different combination of number, aperture and position of the collimators is used to create a high degree of tumor conformality by developing a plan with multiple isocenters, preserving a sharp fall-off in dose to surrounding structures while providing conformal coverage of the irregular target.

#### Linac

**Figure 2:**
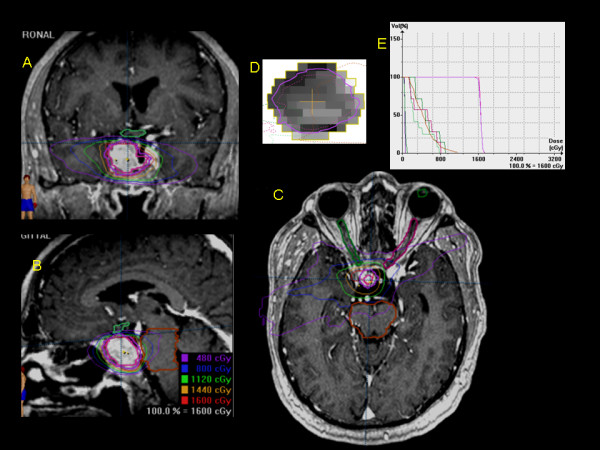
**Prescription isodose distribution in sagittal (A), coronal (B) and axial (C) views of a recurrent pituitary adenoma extending to the parasellar region, and in close proximity of the optic chiasm (contoured in green) and the brainstem (contoured in brown). **The target volume (contoured in purple) was created by the geometric expansion of pituitary tumour plus 1 mm. The patient was treated with a linear accelerator (LINAC) stereotactic radiosurgery (SRS) with a single dose of 18 Gy prescribed to the 95 per cent isodose line. The 90 per cent, 70 per cent and 50 per cent isodose curves showing dose levels delivered to surrounding tissues and adjacent critical structures are represented. High-dose homogeneity and conformity was achieved with the use of intensity-modulated stereotactic radiosurgery (IMSRS), in which intensity modulation of dose for each beam is obtained by moving the leaves in the micro multi-leaf collimator during the course of treatment (**D**). The dose-volume histogram (DVH) graph indicates that doses to optic chiasm (green) and brainstem (brown) were below the tolerance generally accepted for these stuctures (**E**), while delivering an homogeneus dose to the target volume (purple).

Instead of using an array of Cobalt sources, Linac SRS utilizes photons which are derived from colliding accelerated electrons with a metal target. Patients are immobilized in a fixed frame (with less than 0.5-1 mm accuracy) and the treatment is delivered through the use of multiple arcs or beams resulting in a similar high dose differential between the target and normal tissues. Isodose gradients can be improved by the use of intensity modulation of the beams, restriction of gantry angles and arc lengths, microcollimation, and multiple isocenters. SRS may be delivered using cones or circular collimators or, more frequently, micro-multileaf collimator (MLC). MLC consists of a computer-controlled array of up to 120 parallel, individually-adjustable leaves that are attached to the head of the Linac. The leaves of the MLC can be moved in and out to create an adjustable aperture through which radiation beams are directed to the patient’s tumour. The treatment can be delivered as fixed beams or dynamic arcs and the aperture shape can be dynamically changed as treatment progresses. The MLC also facilitates intensity modulated radiosurgery (IMRS), which enables more complex dose distributions for irregular, non spherical tumours. Using the adjustable leaves of an MLC a different dose intensity can be delivered to different areas of the tumour, with the aim to reduce the dose in areas where the beam is close to or is passing through radiation-sensitive brain structures, such as the optic pathways, cranial nerves, and brainstem (Figure 2). Radiation delivered by Linac-based SRS is more homogeneous in dose if compared to GK and this may represent an advantage when treating larger tumours that include radiation-sensitive brain structures. By contrast, GK may achieve a better conformality when irradiating irregularly-shaped targets if compared to Linacs [4].

#### Cyber knife

CyberKnife (Accuray, Sunnyvale, CA) is a relatively new technological device which combines a mobile linear accelerator mounted on a robotic arm with an image-guided robotic system allowing for frameless SRS. Patient position and motion are measured by two diagnostic x-ray cameras and communicated in real time to the robotic arm for beam targeting and patient motion tracking. Patients are fixed in a thermoplastic mask and the treatment can be delivered in one or few fractions achieving the same level of targeting precision as frame-based SRS [5]. CyberKnife can be used for hypofractionated regimens in patients with tumours involving the optic apparatus and patients not suitable for radiosurgery.

#### Protons

Proton beams are delivered through fixed-horizontal-beam rooms or rotational-beam rooms. A dedicated stereotactic intracranial beam line equipment, the STAR system [6], is located at the Massachusetts General Hospital. For the treatment, patients can be positioned on treatment couches or specific chairs and, in general, the steps involved are very similar to those for photon-based SRS. In order to generate the desired dose distribution, two principal categories of beam delivery systems are used: passive systems (scattering) and dynamic systems using scanning magnets (pencil beam scanning).

In the former system, the beam is spread out by the means of one (single scattering) or two (double scattering) scatterers to cover the field cross-section. The use of a range modulator allows spreading out the Bragg peak in depth to cover the target in that direction. Finally, better dose conformation to the target is achieved with patient field-specific hardware. Brass apertures shape the dose according to the outer target boundary while a plastic range compensator tailors the dose in depth at the expense of a certain unwanted full dose proximal to the target. The interaction of protons with these materials produces fast neutrons that can deposit unwanted and worrisome dose to the patient. Worldwide, proton therapy centres with the exception of Paul Sherrer Institute (PSI) in Villigen (SWI), have employed routinely passive scattering until recently. Currently, there are many efforts in developing a more innovative delivery technique, pencil-beam scanning (PBS), in which a narrow pencil beam is magnetically deflected to paint the dose throughout the target. PBS allows the dose conformation laterally, distally as well as proximally to the target volume. Due to the absence of proton interactions with the aforementioned materials the neutron dose to the patient is also minimized. PBS inherently promotes the delivery of intensity-modulated proton therapy (IMPT). In fact, the optimization of energy and intensity of the narrow pencil beams allows the delivery of non-homogenous dose distributions for every single field. The superposition of multiple (inhomogeneous) fields delivers a very homogenous, highly conformal treatment while best sparing organs at risk. It is noteworthy that due to the energy-dependent depth dose distribution IMPT introduces an additional degree of freedom in dose modulation with respect to photon IMRT. At the moment this innovative delivery technique has not been applied to radiosurgical treatments but could deserve further investigation especially in case of regular and irregular shaped, medium to large lesions.

### Radiobiology of SRS

Fractionation is a key strategy in conventional radiotherapy. The corresponding radiobiological effects provide sparing of the normal tissues, while producing optimal tumour damage [7]. Conversely, SRS exploits a different pattern of dose distribution, rather than radiobiological differences between normal and tumour tissue, to achieve effective tumour destruction.

Experimental and clinical data suggest that the radiobiological principles may differ when irradiation is delivered in large single doses [8] or when a different type of radiation such as protons is employed [4].

For a comprehensive review of the radiobiological principles of fractionation and radiosurgery, the reader is referred to specific textbooks and articles [9-11].

An overview on the aforementioned topics is provided as follows.

Firstly, because of their oxygen deficiency, hypoxic cells are highly resistant to the radiation-related killing [12]. The oxygen-related influence is quantified by the so-called oxygen enhancement ratio (OER). Unfortunately, regardless of their size, malignant tumours usually contain a certain amount of hypoxic cells.

Secondly, the dose–response relationship differs according to the type of tissues. Those containing mainly non-cycling cells (“late reacting tissues” [13]), such as most benign tumours and cerebral healthy tissues are less sensitive to small doses per fraction than tissues containing mainly cycling cells (“early reacting tissues” [13]), such as malignant tumours.

Finally, experimental data show that cells have different radiation sensitivities in different parts of the cell cycle [14],

Taking such knowledge into account, the employment of a fractionated regimen allows hypoxic cells to reestablish their oxygenation state [11] so that they will be more sensitive to a second, and subsequent, dose fraction. Dose fractionation also spares late reacting (healthy) tissues more than early reacting (malignant tumour) tissues [10]. Finally, it allows part of the cells to leave the resistant phase while entering in a more sensitive phase. As an overall result, a more effective cell killing takes place.

Conversely, by delivering high single doses, the OER is higher so that such regimen may be disadvantageous if the target tissue is anoxic [9], early responding, and is in close proximity of or embedded within late responding organs at risk [15]. Finally, the delivery of a single large dose does not allow redistribution of cells into a more radiosensitive phase of the cell cycle. However, the rationale for SRS is that the radiobiological effect of a single large dose of radiation results in cell kill or loss of cell division capability, regardless of the mitotic phase.

The advantage of fractionated regimens probably does not apply to benign tumours considering that hypoxia unlikely plays a significant role and both tumour cells and surrounding normal tissues belong to the same radiobiological type [10]. Provided that SRS dose fall-off is steep enough to surrounding structures, the delivery of high single dose to slow growing lesions should translate into a greater rate of local tumour control, while still offering a low rate of complications.

Protons and photons differ in terms of physical properties and interaction with matter, which ultimately translate into different dose distribution as well as biological effectiveness [3]. To date, such difference has been quantified in a 1.1 relative biological effectiveness (RBE) of protons over photons [16]. Although a generic RBE may not reflect slight tissue and dose dependent variations in RBE, these variations are not clinically relevant [1]. As a consequence, all of the above stated radiobiological principles as well as the corresponding clinical applications keep their validity regardless of the employed type of radiation so that there is no difference between photon- and proton-based SRS.

However, experimental data have shown that proton RBE values increase over the last few millimeters of the range, ultimately leading to an increased linear energy transfer. To take into account the uncertainties of the RBE in the terminal 2 mm of each spread-out Bragg peak (SOBP) during proton treatment planning, one could expand of 2 mm or more the distal penumbra [16], which might be clinically relevant for the surrounding healthy structures. From the radiobiological standpoint, this issue probably represents the most relevant difference between photon- and proton-based SRS so that it is wise to take into account the biological effects of this high-RBE component during the planning.

### Dosimetrical features of SRS

Effective radiosurgery is based on the principles of specificity and selectivity [11,17]. Precision and conformal dose planning allow specific target irradiation with a steep fall-off into other surrounding structures. Selectivity refers to the biological differences in the response of different tissues.

The SRS techniques have been compared in several plan comparison studies [18-21]. The corresponding efficacy has been investigated also on the basis of the normal tissue complication probability (NTCP) and tumour control probability (TCP) models in the attempt to set the results also on a biological basis [20-22].

In general, the difference among photon-based SRS techniques (GK, multi- non-coplanar arcs or shaped beams linac treatment) are negligible [18-21] even though GK may confer greater dose heterogeneity. Conversely, the modality (photons or protons) can result more important. Target features such as size, shape and location within the brain can influence the choice for the best SRS modality. In fact, all modalities are equally good if the target is small and regular [20,21].

Based on the normal brain dose, the dosimetrical advantage of charged particles relative to photons is evident in all types of target [18,20,21]. Such difference is more relevant under the 60 per cent dose level regardless of the target features [20]. Moreover, the larger the target volume the greater the difference [18,20,21], which peaks for regular shaped targets larger than 24–26 cc even though it can be relevant even for smaller and irregular targets (about 6 cc) [20,21].

All of the above-stated considerations also apply with respect to the lesion’s shape and location.

In case of photons, both GK and shaped beams Linac fields allow for better target conformality [19,21] when compared to standard arcs with fixed circular collimators Linac treatments. A further consideration can be made regarding the dose within the target. In fact, if the area to be irradiated includes normal brain tissue, the target dose homogeneity represents a further parameter that can reduce the risk of side effects. Dosimetrical comparisons point out that protons have an advantage over other modalities, particularly in the case of irregular targets [21]. However, it is unknown if greater homogeneity can influence tumour control rates in SRS.

All of the above-mentioned quantitative differences were confirmed when the analysis was approached on a biological basis, being the NTCP different according to the treatment modality, size, shape and location of the target [20,22]. Again, protons demonstrated the lowest NTCP for medium-large regular and irregular shaped lesions [20,22]. In this scenario, charged particles scored NTCP values 4–6 per cent smaller than photon techniques.

In this context, it is noteworthy that radiation-induced tumours were reported after photon SRS [23,24]. It is well-known that protons feature a low integral dose to healthy structures providing the potential to reduce this risk. However, the tissue volume that can benefit from this feature may be very small in SRS and the corresponding clinical gain might be difficult to detect.

In conclusion, in the attempt to customize the treatment according to the clinical scenario, it is possible to state that small to medium regularly shaped lesions can be effectively managed by all photon-based techniques although at the expense of some target dose inhomogeneity. The charged particle capability to simultaneously provide high target conformity and dose homogeneity maximizes for regularly- and irregularly-shaped, medium to large lesions.

Finally, it is noteworthy that despite such comparisons included several planning and treatment strategies, further improvements, such as IMRT and IMPT have been introduced. These certainly deserve further investigation.

### Clinical results of photon and proton SRS for skull base tumours

Search strategy and selection criteria

Data for this review were obtained using MEDLINE databases, which were searched for publications between period of January 1980 and December 2011.

The search terms were: “skull base” and “stereotactic radiosurgery”. Further search was made by adding the definitions of different SB tumours (“meningioma”, “schwannoma/acoustic neuroma”, “pituitary adenoma”, “chordoma”, “chondrosarcoma”, “craniopharyngioma”, “olfactory neuroblastoma/esthesioneuroblastoma”, “glomus jugulare/chemodectoma”, and “proton”) to the previously-searched keywords.

The search was limited to articles written in English. Editorials, case reports, letters of opinion, and congress abstracts were excluded, even if they added valuable information. In case of repeated publications from the same institution, only the most updated was used for the analysis. Papers of the search were reviewed and prioritized according to content relevancy. Reference lists from these sources were searched for additional publications. A systematic review was beyond the aim of the paper; the following results are reported in the form of a narrative synthesis.

## Results

In total, 449 reports related to SRS for base of the skull tumours were retrieved from the initial PubMed and reference lists search: 261 from the first search and 188 from the following. In particular, 104 studies regarded meningioma, 26 schwannoma/acoustic neuroma, 19 pituitary adenoma, 11 chordoma and/or chondrosarcoma, 6 craniopharyngioma, 3 olfactory neuroblastoma/esthesioneuroblastoma, 7 glomus jugulare/chemodectoma. After applying the inclusion criteria, 298 studies were considered for this review.

SRS has been increasingly employed as primary or post-operative treatment with more than 10,000 patients reported in published studies over the last two decades. Much less data is available on the treatment with proton-SRS (nine reports) even though several types in benign and malignant settings and also non-tumoural lesions as arteriovenous malformations have been treated since its early use showing that this is a viable option for larger volumes.

To date, no randomized or non-randomized study has compared photon-SRS with proton-SRS, and almost all the studies available in literature are retrospective*.*

### Meningioma

The majority of meningiomas (90–95 per cent) are benign (WHO grade I). Surgical resection is the preferred treatment for easily accessible tumours that can be safely removed. SRS has been used as an alternative to surgical resection for poorly accessible lesions, as a primary therapy for benign meningiomas or recurrent tumours, and as an adjuvant treatment for post-surgical residual lesions. The 5-year control rate is equivalent to that of gross-total resection but with lower morbidity. Additionally, adjuvant treatment of subtotally resected tumours results in local control rates equivalent to gross-total resection [25].

Recently published multicenter series and reviews [26-30] on benign lesions show a 5-year control rate ≥ 92 per cent. SRS is usually reserved for locations such as the cavernous sinus where surgical risk is expected to be higher and is considered suitable for meningiomas with maximum diameter less than 3 to 4 cm, with distinct margin with minimal to no surrounding oedema and with sufficient distance from critical normal tissue to allow for appropriate normal tissue dose restriction [29,30]. Radiosurgical doses between 12 and 18 Gy have been used in the control of SB meningiomas. A similar 5-year actuarial tumour control rate in the range of 90–95 per cent has been observed with doses of 15–16 Gy or 12–14 Gy. However, several studies demonstrated that tumour control is decreased for superficial lesions and with increasing tumour size [26,31,32]. In addition, radiation toxicity increases with increasing tumour size and superficial location. After radiosurgery, better outcomes were observed for those receiving an optimal radiosurgery dose and harboring tumours located in a cerebellopontine angle, parasellar, or petroclival location [28].

Radiation-induced toxicity has been shown in up to 40 per cent after SRS, being represented by either transient or permanent neurological complications; however, the reported rate of significant complications at doses of 12–15 Gy, as currently used in most centres, is relatively low. Kondziolka et al. [31] reported permanent neurological deficits of 6·3 per cent for cavernous sinus meningiomas treated with GK SRS. Nicolato et al. [33] showed late transient or permanent complications in 4,5 per cent of patients, and similar complication rates have been reported in the majority of published series [28]. Other complications, as epilepsy, internal carotid occlusion, and hypopituitarism have been rarely reported (less than 1–2 per cent).

Atypical or malignant meningioma are usually irradiated adjuvantly after complete surgical excision. EBRT is usually employed after surgery whereas SRS is reserved to recurrences; the experience in this field is, however, very limited [34].

Protons have usually been used in this context with conventional fractionation and in association with photons [35-37], but also with hypofractionated regimens or single-session SRS [38-40]. Proton HSRT with three or more fractions showed a local control of 91 per cent at 5 years [38] and 100 per cent at 3 years [39]. The group of MGH in Boston [40] has recently reported the results of 51 cases of benign meningioma treated with proton-SRS between 1996 and 2007 as primary treatment (n = 32) or for residual tumour following surgery (n = 8), or recurrent tumour following surgery (n = 10). The median dose delivered was 13 Gy (RBE) (relative biological effectiveness) (range, 10 -15·5 Gy (RBE)) prescribed to the 90 per cent isodose line. After a median follow-up of 32 months (range, 6–133 months), MRI revealed 33 meningiomas with stable, 13 with decreased, and five with increased size. The 3-year actuarial tumour control rate was 94 per cent. Symptoms were improved in 47 per cent (16/34) of patients. Potentially permanent adverse effects after SRS were recorded in 3/51 (5·9 per cent) patients. The main limitation of these studies is that longer follow-up is needed to assess the durability of tumour control.

### Pituitary adenoma

The role of RT in pituitary adenomas is well-established [41] particularly when medical and surgical options have been exhausted. Therapeutic goals when performing RT for pituitary tumours are: 1) stopping of tumour growth by preventing problems from mass effect and 2) normalize excessive hormone secretion.

All main published results on the long-term effectiveness of SRS in patients with nonfunctioning and secreting pituitary adenomas have been recently reviewed [42,43]. In 15 studies reporting 684 patients with non-functioning adenomas treated with SRS at doses of 15–22 Gy, the reported 5-year actuarial tumour control rate was 94 per cent. A similar local control was observed in patients with secreting pituitary adenomas, although higher doses in the range of 18–26 Gy were employed with the aim to achieve normalization of hormone hypersecretion. SRS data for 1215 patients with acromegaly have been reported in 29 studies [42]. At a median follow-up of 50 months, the 5-year and 10-year biochemical remission rates were 44 per cent (range, 15–60 per cent) and 74 per cent (range, 46–86 per cent), respectively. Time to response ranged from 12 to 66 months. Results of SRS have been reported for 280 patients with Cushing’s disease in 12 studies [43]. At a corrected median follow-up of 45 months, 48 per cent of patients had biochemical remission of disease, with a reported time to hormonal response ranging from 3 months to 3 years. SRS is rarely used in the treatment of prolactinomas since medical treatment with dopamine agonists can achieve tumour shrinkage and normalize prolactin (PRL) levels in more than 80 per cent of patients. When employed in patients who fail surgery and medical therapy, at a median follow-up of 29 months, normalization of elevated PRL levels has been observed in 33 per cent of 353 patients included in 18 studies, with a reported time to hormonal response ranging from 5 to 40 months [43]. The reported overall rate of serious complications after SRS is low. The main complication is hypopituitarism which is reported in up to 47 per cent of patients, with higher rates in those series with a longer median follow-up.

Data on proton treatment in pituitary adenomas are available both with the option of conventional fractionation [44] and with SRS [45,46]. In a small series of 22 patients treated with proton SRS for persistent acromegaly at a median follow-up of 6·3 years, the biochemical remission of disease was observed in 13 patients (59 per cent) [45]. Time to response was 42 (range, 6–62) months. In a retrospective series of 33 patients with Cushing’s disease at a median follow-up of 62 months, normalization of plasma and urinary free cortisol was achieved in 17 (52 per cent) patients, with a time to remission of 18 (range, 5–49) months [46]. In both series the only reported toxicity was represented by new pituitary deficits which occurred in up to 52 per cent of patients, whereas no visual complications, seizures, or secondary tumours were noted. The small number of cases treated and limited follow-up precludes to draw firm conclusions. Proton-SRS may offer better dosimetric coverage of the pituitary gland than photon-based treatments that could be particularly useful in paediatric patients [47,48].

### Acoustic neuroma/vestibular schwannoma

Acoustic neuroma is the most studied disease to which SRS is applied as a stand alone treatment. SRS as an effective treatment for acoustic neuroma has evolved over the last decades, leading to an improvement of local control and reduction of long-term toxicity. At doses of 12–13 Gy, as used in most recent studies, SRS results in an actuarial 5-year tumour control between 92 and 100 per cent with a low incidence of radiation-induced complications [49]. Current evidence supports its use for small to medium sized primary and recurrent vestibular schwannomas. It is also recommended for adjunctive therapy, recurrent tumours, in poor surgical candidates, and for those who do not desire observation or surgery [50].

The reported local control with doses of 12–13 Gy is similar to that reported with higher doses in the range of 15–18 Gy as used in early experiences of SRS, however with a lower incidence of radiation-induced complications. A recent review of more than 2000 patients included in 23 studies has shown an overall facial nerve preservation rate of 96 per cent after GK, with a significant better facial nerve preservation rate in patients receiving ≤ 13 Gy of radiation at the marginal dose and with a tumour volume ≤ 1·5 cm^3^[51] . Using similar doses, an overall hearing preservation, as defined by the maintenance of Gardner-Robertson Grade I or II after SRS, has been reported in 51 per cent (range, 32–71 per cent) of 4234 patients included in 45 publications [52]. Equivalent tumour control and hearing preservation rates have been reported for larger acoustic neuromas compressing the brainstem, with a reported balance improvement or stabilization in more than 85 per cent of patients who had imbalance at presentation [53]. Neurological toxicity, including facial and trigeminal neuropathies, and balance disturbances may occur in 0–3 per cent of patients. Hydrocephalus has been observed in 1–2 per cent of patients, whereas radiation-induced tumours or malignant transformation of acoustic neuroma have been reported rarely [50-54].

Also in this site, proton beam has been used with conventional fractionation [55] or with SRS with a satisfactory level of hearing, facial nerve, trigeminal nerve preservation, and with tumour control rates of 84–100 per cent [56-58]^.^ Weber et al. reported on 88 patients treated at the MGH between 1992 and 2000 with proton-SRS [56]. At a median follow-up period of 38,7 (range, 12–102) months the actuarial 2- and 5-year tumour control was 95,3 per cent and 93·6 per cent, respectively. Hearing was preserved in 33 per cent of 21 (24 per cent of the total) patients with functional hearing before treatment. Actuarial 5-year normal facial and trigeminal nerve function preservation rates were 91 per cent and 89 per cent, respectively. Three patients (3,4 per cent) underwent shunting for hydrocephalus.

HSRT with a dose of 26 Gy (RBE) in three fractions was delivered with protons in the study of Verminnen et al. [58]. At a median follow-up of 72 months, the 5-year local control was 98 per cent.

### Craniopharyngioma

The treatment of craniopharyngioma is based on a surgical treatment with transcranial approaches or endoscopic endonasal surgery followed by RT, mainly in form of fractionated regimens with a local control of 80–90 per cent at 5–10 years [59]. The proximity of craniopharyngiomas to the optic pathways provides a major limitation to the use of SRS, although in very selected series of relatively small residual tumours, a local control rate between 34 and 88 per cent has been reported [60-62]. Tumour control was achieved with a median dose of 22–24 Gy (marginal dose 11–12 Gy), whereas the use of lower radiation dose results in an unsatisfactory tumour control [63]. The reported late toxicity after SRS ranges from 0 to 38 per cent, mainly represented by visual and endocrinological deficits [59]. In general, treatment of craniopharyngiomas with SRS is not considered a standard procedure.

Protons have been recently used for the treatment of this tumour but only with fractionated regimens [64], and to date no experience with proton SRS has been reported.

### Chordoma – chondrosarcoma

Nowadays, the improvements in surgical techniques allow more radical resection of these tumours, while frequently providing small residual lesions, which can be suitable for SRS. Again, very few clinical data have been published on this issue with promising preliminary results showing that SRS at marginal doses of 14–16 Gy could represent a valuable treatment option for small-sized chordomas residual after surgery or relapsing [65-68].

Data on this issue are limited and SRS cannot be considered a standard form of treatment for these tumours.

Protons have been used only with conventional fractionation at doses ≥ 70 Gy both in chordoma and chondrosarcoma, even of large volume, with very satisfying results independently by their size [69,70].

No data are available at this moment with the use of proton-SRS.

### Chemodectoma/glomus jugulare tumours

Radiation has been found to be helpful in controlling glomus jugulare tumour growth by inducing fibrosis around the supplying vessels. In 2011, a comprehensive search of the English-language literature identified 109 studies that collectively described outcomes for patients with glomus jugulare tumours [71]. Data collected from 869 patients were assessed. Patients undergoing SRS had the lowest rates of recurrence and the most favourable rates of tumour control. In particular, those treated with subtotal resection plus SRS had a control rate of 71 per cent at 96 months of follow-up. Patients undergoing SRS alone at a median follow-up of 71 months had a tumour control rate of 95 per cent. A recent meta-analysis [72] was published on these tumours treated with SRS. The data search yielded 19 studies. Across all studies, 97 per cent of patients achieved tumour control, and 95 per cent of patients achieved clinical control suggesting considering SRS for the primary management of glomus jugulare tumours. Average marginal dose in Gy delivered was between 12 and 20.4 Gy (median 15 Gy, mean 15.17). In particular, SRS can be considered a safe and effective treatment for patients with preserved glossopharyngeal and vagus nerve function, after surgical recurrence, in the elderly, and in patients with serious preexisting medical conditions [73].

Again, no data are available for the use of proton-SRS in this field.

### Olfactory neuroblastoma/esthesioneuroblastoma

Olfactory neuroblastoma or esthesioneuroblastoma is a rare tumour of the frontal SB still associated with high rates of tumour recurrence and mortality. A meta-analysis by Dulguerov and colleagues [74] demonstrated that surgery with radiation is the most frequently used therapeutic approach, and the one achieving the highest cure rates. Also, in this disease SRS cannot be considered standard of care. The data in literature is scarce: radiosurgery can be considered in combination with endoscopic sinus surgery as a promising treatment option [75,76].

## Conclusions

Stereotactic radiosurgery is an increasingly-used treatment option supported by extensive number of studies in the management of SB tumours. Current data show excellent results in treating several tumours of the SB including meningioma, pituitary adenoma and vestibular schwannoma. Long-term data indicate a tumour control in more than 90 per cent of cases after 5 and 10 years, with an acceptable incidence of complications. Favourable data with the use of SRS are reported also for other rare tumours such as craniopharyngioma, chordoma, olfactory neuroblastoma and glomus jugulare tumour. Further studies are needed in order to fully elucidate its role in these lesions while considering the high risk of radiation injury to tissues.

In most series, GK is the most widely used radiosurgical technique although the reported outcome is similar for patients with SB tumours treated with other photon techniques.

Even though proton therapy is increasingly used in the clinical community, only few studies have been performed to assess the efficacy and toxicity of proton-SRS in SB tumours. The number of institutions that are currently using protons is still small, particularly those performing proton-based stereotactic techniques.

Proton beam, while utilizing a different form of radiation, also represents a similarly highly focused and targeted radiation tool. Even though the physical properties of protons could offer superior conformality in dose distribution with respect to photons, current clinical results show a similar control for such tumours because similar doses were used across photon and proton experiences. Proton SRS has been advocated as preferred treatment of larger and more complex SB lesions. Current data do not allow any definitive conclusion about their presumed superiority over other photon-based SRS techniques mainly because of the limited number of patients treated with protons. This makes it difficult to compare the results. In respect to the small number of patients treated with proton SRS and short follow-up, toxicity was similar with the use of the different techniques; however, the evaluation of complications is often completely subjective and unsatisfactory. It is important to emphasize that protons were used with the aim to minimize normal tissue toxicity rather than increase local control. The difference between techniques may be quite small and large numbers of patients, followed for long periods of time, would be needed in order to demonstrate any clinically significant advantage. However, given the still-high costs of protons [77], comparative multi-institutional trials are needed to select the appropriate modality for each tumour type.

## Abbreviations

SRS: Stereotactic radiosurgery; SB: Skull base; GK: Gamma Knife modified; Linac: Linear accelerators; EBRT: External beam radiation therapy; HSRT: Hypofractionated stereotactic radiation therapy; MLC: Multi-leaf collimator; IMRS: Intensity modulated radiosurgery; PBS: Pencil-beam scanning; IMPT: Intensity-modulated proton therapy; RBE: Relative biological effectiveness; OER: Oxygen enhancement ratio; SOBP: Spread-out Bragg peak; NTCP: Normal tissue complication probability; TCP: Tumour control probability; Gy: Gray; PRL: Prolactin.

## Competing interest

We declare that we have no conflicts of interest

## Authors’ contributions

All authors specify that they made substantive intellectual contributions contributing equally to the review. In particular AM, DA and GM made substantial contributions to conception of the review and interpretation of data, have been involved in drafting the manuscript, and have given final approval of the version to be published. All authors read and approved the final manuscript.

## Aknowledgements

We thank Valentina Piffer (ATreP, Trento – Italy) for her language editing of the manuscript.
